# Efficacy and safety of ruxolitinib for steroid-refractory graft-versus-host disease: Systematic review and meta-analysis of randomised and non-randomised studies

**DOI:** 10.1371/journal.pone.0271979

**Published:** 2022-07-29

**Authors:** Meng-yun Zhang, Peng Zhao, Yan Zhang, Ji-shi Wang

**Affiliations:** 1 School of Clinical Medicine, Guizhou Medical University, Guiyang, GuiZhou Province, China; 2 Department of Haematology, The Affiliated Hospital of Guizhou Medical University, Guiyang, GuiZhou Province, China; University of Missouri, UNITED STATES

## Abstract

**Background:**

Hematopoietic stem cell transplantation (HSCT) for haematological disorders. Graft-versus-host disease (GVHD), a cause of morbidity and mortality is treated with corticosteroids. However, patients with steroid-refractory GVHD after HSCT have a poor prognosis. Ruxolitinib, a selective Janus kinase inhibitor, is a novel treatment strategy for steroid-refractory GVHD.

**Objectives:**

To assess the efficacy of ruxolitinib for the treatment of steroid-refractory GVHD and analyse its adverse effects.

**Study design:**

Meta-analysis.

**Search methods:**

Randomised controlled trials (RCTs) and non-RCTs of ruxolitinib-based therapy in patients with steroid-refractory GVHD were found in the Cochrane Central Register of Controlled Trials, EMBASE, PubMed, and Web of Science in March 2021. Outcomes included overall response rate, survival, and adverse effects. The Methodological Index for Non-randomised Studies (MINORS) and the Cochrane collaboration risk-of-bias tool were used to assess methodological quality. Funnel plots, Egger’s test, and the trim and fill method were used to assess publication bias.

**Results:**

In total, 1470 studies were identified; 19 studies (17 non-RCTs, 2 RCTs) involving 1358 patients met our inclusion criteria. Survival rates at the longest follow-up in non-RCTs, were 57.5% (95% CI 46.9–67.4) and 80.3% (95% CI 69.7–87.9) for acute GVHD (aGVHD) and chronic GVHD (cGVHD), respectively. In non-RCTs, the overall response was 74.9% (95% CI 66.6–81.8, I^2^ = 49%) in aGVHD and 73.1% (95% CI 62.5–81.6, I^2^ = 49%) in cGVHD. In aGVHD, the response rates were gastrointestinal, 61.4–90.2%; skin, 52.5–80.6%; and liver, 41.8–71.8%. In cGVHD, the response rates were gastrointestinal, 30.1–70.4%; skin, 30.1–84.4%; lung, 27.0–83.0%; and mouth 3.5–98.1%. In addition, a lower aGVHD grade and moderate cGVHD were associated with a better clinical response. Common adverse events were cytopenia and infectious complications.

**Conclusions:**

Our systematic review and meta-analysis indicated that ruxolitinib therapy could be a potentially effective and safe treatment for patients with steroid-refractory GVHD.

## Introduction

Haematopoietic stem cell transplantation (HSCT) is used to treat many malignant and non-malignant haematological disorders [1]. Graft-versus-host disease (GVHD) is a serious complication after HSCT that causes morbidity, mortality, and has a tremendous impact on the quality of life [2, 3]. In recent years, the number of patients experiencing GVHD has increased to 70% [4]. GVHD is a clinical syndrome caused by donor-mediated immune reaction in HCT recipients. Proinflammatory cytokines such as IL-1, IL-6, tumour necrosis factor (TNF)-α, and interferon (IFN)-γ are associated with GVHD [5]. Clinically, acute (aGVHD) and chronic (cGVHD) are classified according to the time of occurrence after transplantation, many organ systems including skin, gastrointestinal tract, liver, lung, mouth, oesophageal, musculoskeletal, genital tissues, joint and eyes are involved in this category [4, 6]. Strategies for the treatment of aGVHD and cGVHD primarily focus on standard first-line treatment (corticosteroids) [7]. However, steroid-refractory GVHD (SR-GVHD) occurs in half of all patients with GVHD after allogeneic haematopoietic cell transplantation and has a poor prognosis. Second-line therapies for SR-aGVHD include alemtuzumab, α1-antitripsin, basiliximab, cellular therapies (mesenchymal stem cells (MSC) or regulatory T cells), daclizumab, extracorporeal photopheresis (ECP), faecal microbiota transplantation, Janus kinase (JAK) inhibitors, mycophenolate mofetil, methotrexate, pento statin, rabbit thymoglobulin, sirolimus, and vedolizumab [7]. For SR-cGVHD, second-line therapies include calcineurin inhibitors, ECP, ibrutinib, JAK inhibitors, mycophenolate mofetil, rituximab, mammalian target of rapamycin inhibitors, pentostatin, proteasome inhibitors, and tyrosine kinase inhibitors [7, 8].

The choice of standardised second-line therapy for SR-GVHD is controversial. Ruxolitinib, a selective (JAK1 and JAK2) inhibitor, reduces GVHD by inhibiting the production of proinflammatory cytokines, including IL-1, IL-6, IL-12, IL-17, TNF-α, and IFN-γ, reducing T-cell proliferation and preserving the beneficial graft-versus-leukaemia effect [9–12]. In a retrospective, multicentre study involving 54 patients with SR-aGVHD, Zeiser et al. [13], reported an overall response rate (ORR) of 81.5% in patients treated with ruxolitinib, including a 46.3% complete response. The phase 2, single-arm ruxolitinib for the treatment of steroid-refractory acute GVHD (REACH1) study [14] has been encouraging, with an ORR at day 28 of 54.9%. The overall survival at 6 months was 51%. Based on these studies, the US Food and Drug Administration approved ruxolitinib for the treatment of SR-aGVHD.

In summary, these potential mechanisms make ruxolitinib an advantageous second-line therapy in cases of SR-GVHD. Because of the importance of ruxolitinib as a possible novel strategy to treat patients with SR-GVHD, we performed a systematic review and meta-analysis of the available data to provide evidence for the efficacy of ruxolitinib in the treatment of GVHD.

## Materials and methods

### Search strategy

We searched the following electronic databases: Cochrane Central Register of Controlled Trials, PubMed, EMBASE, and Web of Science, to identify prospective and retrospective studies evaluating the efficacy of ruxolitinib in patients with SR-GVHD. The search was conducted in May 2021. Studies were limited to all publications up to that time. The search specifics were as follows: (“Graft vs Host Disease” [Mesh] OR “graft versus host*” Title/Abstract [TIAB] OR “graft vs host*” [TIAB] OR “graft v host*” [TIAB] OR “gvhd” [TIAB] OR “runt diseas*” [TIAB], OR “homolog* wasting diseas*” [TIAB]) AND (“Janus Kinase 1” [Mesh] OR “Janus Kinase 2” [Mesh], OR “jak1*” [TIAB] OR “jak2*” [TIAB] OR “Ruxolitinib*” [TIAB] OR “jakafi*” [TIAB] OR “jakavi*” [TIAB] OR (“jak*” [TIAB] and “inhibit*” [TIAB]) OR (“janus*” [TIAB] and “kinas*” [TIAB]) OR “INCB018424” [TIAB] OR “INCA24” [TIAB]). The results were limited to human studies and those published in English, the search criteria were wide, and all possible relevant articles were available.

### Inclusion/Exclusion criteria

All studies included in this meta-analysis met the following criteria: (1) All patients had haematological diseases. (2) Studies on patients with SR-GVHD after HSCT without age limitations. (3) Use of ruxolitinib. (4) Ten or more patients were enrolled. (5) Studies published in English Review studies, case reports, animal models, cell lines, letters, duplicate publications, and meeting or conference abstracts without available data were excluded.

### Data extraction and quality assessment

Two authors independently extracted the data by collecting the following information: the first author, published year, characteristics of patients (age), initial ruxolitinib dose, graft source, type of GVHD, conditioning regimen, and outcomes (overall survival, overall response, complete and partial response, and organ-specific response). Following data extraction, we assessed the possible risk of bias of randomised-control trials (RCTs) using the Cochrane Collaboration risk-of-bias tool, where each included trial was scored as low, unclear, or high risk of bias. We used the first eight items of the Methodological Index for Non-randomised Studies (MINORS) to assess the methodological quality (risk of bias) of non-RCTs. According to the evaluation items, the total MINORS score ranges from 0 to 16. A score of 0–9 indicates low quality, while 10–16 indicates high quality, and differences were resolved through discussion and consultation.

### Outcomes

All studies reported the response rate, and some reported the survival rate. The primary endpoint for analysis was survival after ruxolitinib treatment. The secondary outcomes of interest were overall response (complete and partial responses), organ-specific responses, and adverse effects.

### Statistical analysis

Data analyses were performed using Comprehensive Meta-analysis version 3.3, and Review Manager software (v 5.4; Nordic Cochrane Centre, Cochrane Collaboration). Compared with the fixed-effects model, the random-effects model combines inter-group heterogeneity with intra-group heterogeneity. We used a random-effects model to pool the outcomes and estimate variance across studies. Statistical significance less than 0.1 was considered significant. All reported P-values were obtained from two-sided tests. Statistical heterogeneity was assessed using I^2^ statistics. Values of 25% indicate mild, 50% moderate, and 75% high heterogeneity. When the p-value of Cochran’s Q test was <0.10, and the I^2^ statistic was >50%, there was statistically significant heterogeneity among the studies. To analyse heterogeneity, we performed sensitivity analyses of the clinical responses and overall survival in patients treated with ruxolitinib for SR- GVHD. Funnel plots, Egger’s test, and the trim and fill method were used to assess publication bias.

## Results

**Fig 1:**
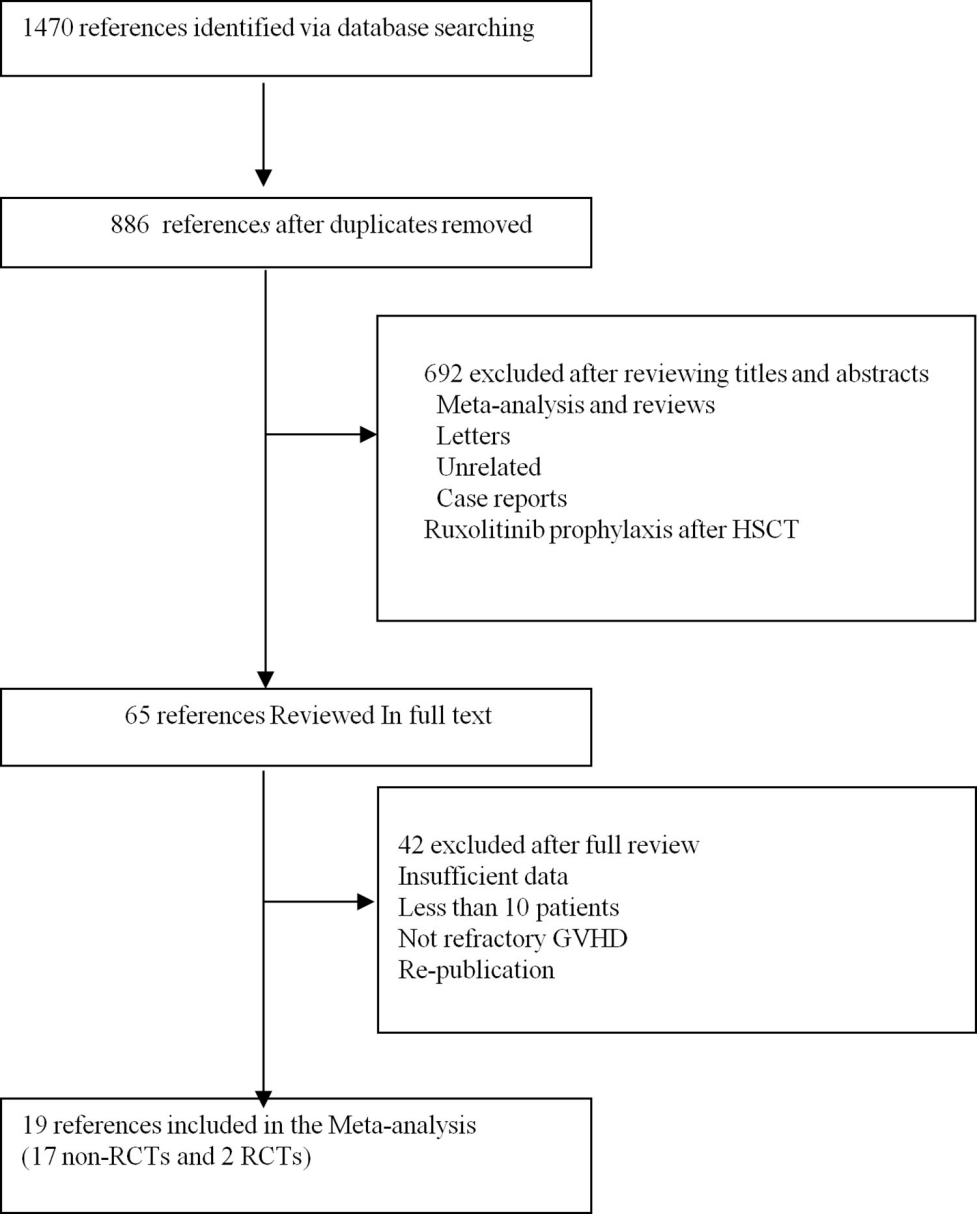
Identification and selection of studies.

A systematic search of the Cochrane Central Register of Controlled Trials, Web of Science, PubMed, and EMBASE included 1470 studies. After removing duplicates, two authors independently screened the titles and abstracts of the selected studies to identify potential studies for inclusion. A full-text review was sought for assessment if the data were not satisfactorily obtained from the title and abstract. Finally, 19 studies (17 non-RCTs [13–29] and two RCTs [30, 31]) involving a total of 1358 patients met our inclusion criteria were selected. The age of the patients ranged from 0 to 77 years. The sample size ranged from 10 to 329. Fig 1 shows the flow chart of the study selection, and the baseline characteristics of the studies included in this systematic review are shown in Table 1. The results of the risk of bias in Non-RCTs are shown in Table 2.

**Table 1:** Clinical characteristics of included studies of ruxolitinib evaluating survival, responses in SR-GVHD.

Study; year	Age, year	Patients with aGVHD, n	Patients with cGVHD, n	Donor type	Conditioning Regimen	graft source	Dose of ruxolitinib	ORR in aGVHD, n	ORR in cGVHD, n
Assouan (2018)	26–65	8	0	MRD;MUD	RIC	NA	10 mg bid	5	0
Meng (2020)	8–38	12	0	MRD	MAC	PBSC	5–10 mg bid	12	0
Khandelwal (2017)	1.6–16.5	11	0	MRD;MUD	NA	PBSC, BM, UCB	2.5–5 mg bid	5	0
Mozo (2021)	0.8–18.1	8	12	MRD;MUD; Haploidentical	RIC;MAC	PBSC, BM, UCB	2.5–5 mg bid	7	11
Ferreira (2018)	23–68	0	20	MRD;URD; Haploidentical	RIC;MAC	NA	5-10mg bid	0	15
Laisne (2020)	0.6–14.5	29	0	NA	TBI+; TBI-;	PBSC;BM; PBSC B+BM;UCB	6.3 mg/m2/d—28.7 mg/m2/d	21	0
Uygun (2020)	0.3–17.5	13	15	MSD;MRD;MUD;Haplo	MAC	PBSC, BM; BM+PBSC	2.5–5 mg bid	11	12
Dang (2020)	14–55	10	28	MRD;MMRD;URD	NA	PBSC, BM; BM+PBSC	5–10 mg bid	10	22
Wu (2020)	17–56	0	41	MRD;Haplo	RIC;MAC	PBSC	NA	0	30
Abedin (2019)	45–70	19	24	MRD;MUD;Haplo;MMUD	RIC;MAC	PBSC, BM	5–10 mg bid	17	19
Modi(2019)	21–77	0	46	MUD;MMUD;Sibling	RIC;MAC;unknown	NA	5 mg bid	0	22
White (2019)	52 (median age)	0	35	NA	NA	NA	10–15 mg daily	0	12
Zeiser (2015)		32	20					27	16
Jagasia (2020)	18–73	71	0	MRD;MUD;MMRD;MMUD; Other	MAC;NMAC; missing	PBSC, BM; UCB	5 mg bid	39	0
Moiseev (2020)	1–67	32	43	MRD;MUD;Haplo	MAC;other	PBSC, BM;	0.15 mg/kg or 10 mg bid	24	35
Gómez (2020)	0–73	23	56	MRD;URD; Haploidentical;	RIC;MAC	PBSC, BM; UCB	Median dose 20 mg/day	16	32
Zeiser (2015)	21–75	54	41				5–10 mg bid	44	35
Zeiser (2020)	12–73	154 VS 155	0	URD;RD	RIC;MAC; NMAC	PBSC, BM; Single cord blood;	10 mg bid	96 vs 61	0
Zeiser (2021)	12–76	0	165 vs 164	URD;RD	RIC;MAC; NMAC	PBSC, BM; Single cord blood;	10 mg bid	0	83 vs 43

BM, bone marrow; UCB, umbilical cord blood; PBSC, peripheral blood stem cell; MRD, matched related donor; MSD, matched sibling donor; MUD, matched unrelated donor; MMUD, mismatched unrelated donor; Haplo, haploidentical; RIC, reduced intensity conditioning regimen; MAC, myeloablative conditioning; TBI, total body irradiation; NA, not available; ORR, overall response rate; GVHD, graft-versus-host disease.

**Table 2:** MINORS evaluation form for non-randomized clinical trials included in the literature.

Study	Assouan (2018)	Meng (2020)	Khandelwal (2017)	Mozo (2021)	Ferreira (2018)	Laisne (2020)	Uygun (2020)	Dang (2020)	Wu (2020)	Abedin (2019)	Modi (2019)	White (2019)	Zeiser (2015)	Jagasia (2020)	Moiseev (2020)	Gómez (2020)	Zeiser (2015)
score	8	10	9	10	8	10	10	10	10	9	10	10	10	10	10	10	10

**Fig 2:**
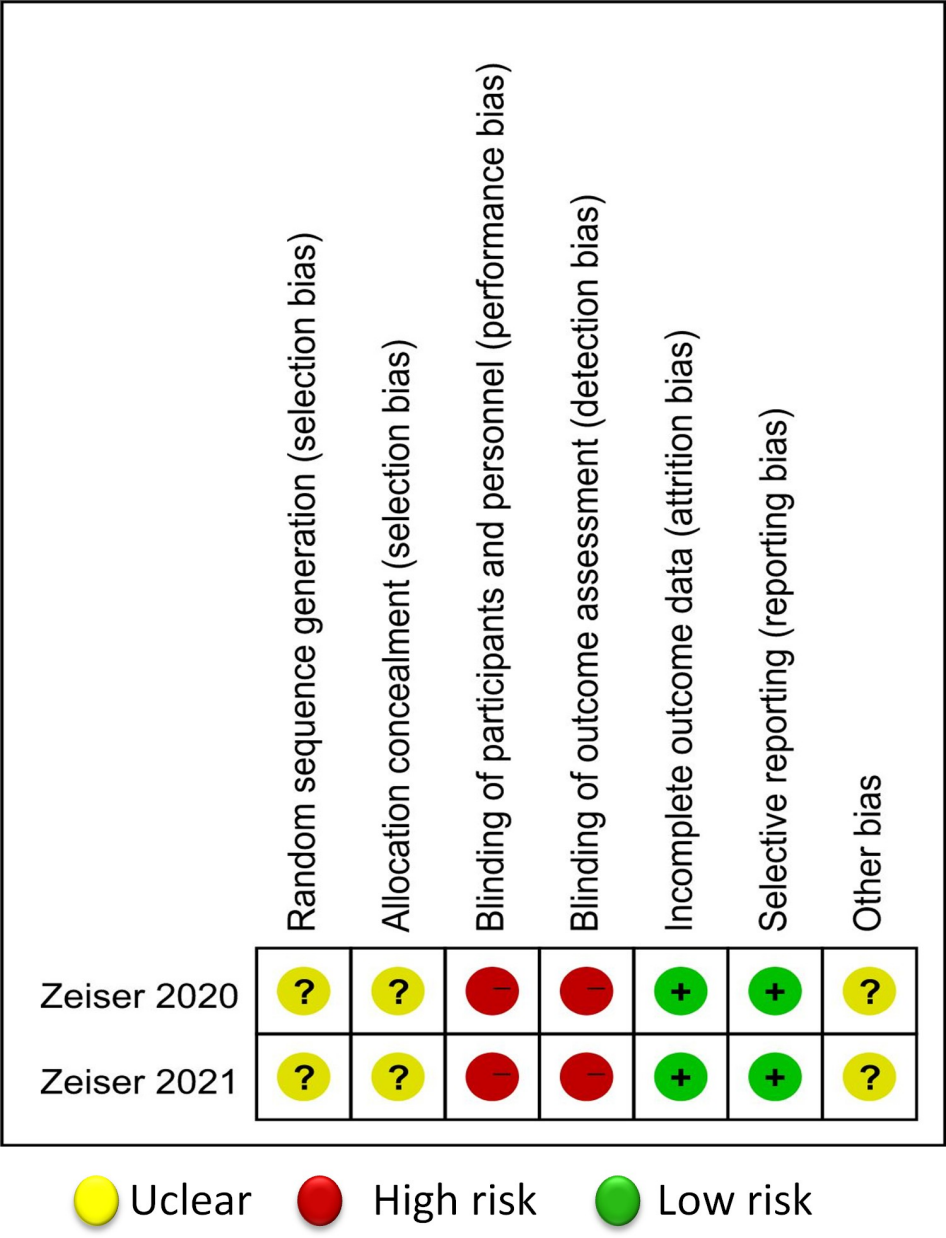
Summary of the risk-of-bias assessment for RCTs.

Two randomised phase 3 trial with a total of 638 participants evaluated the efficacy of ruxolitinib versus best available treatment (BAT) for treating SR-GVHD. The results of the risk of bias in the RCTs are summarised in Fig 2.

### Outcomes

#### Overall survival

**Fig 3:**
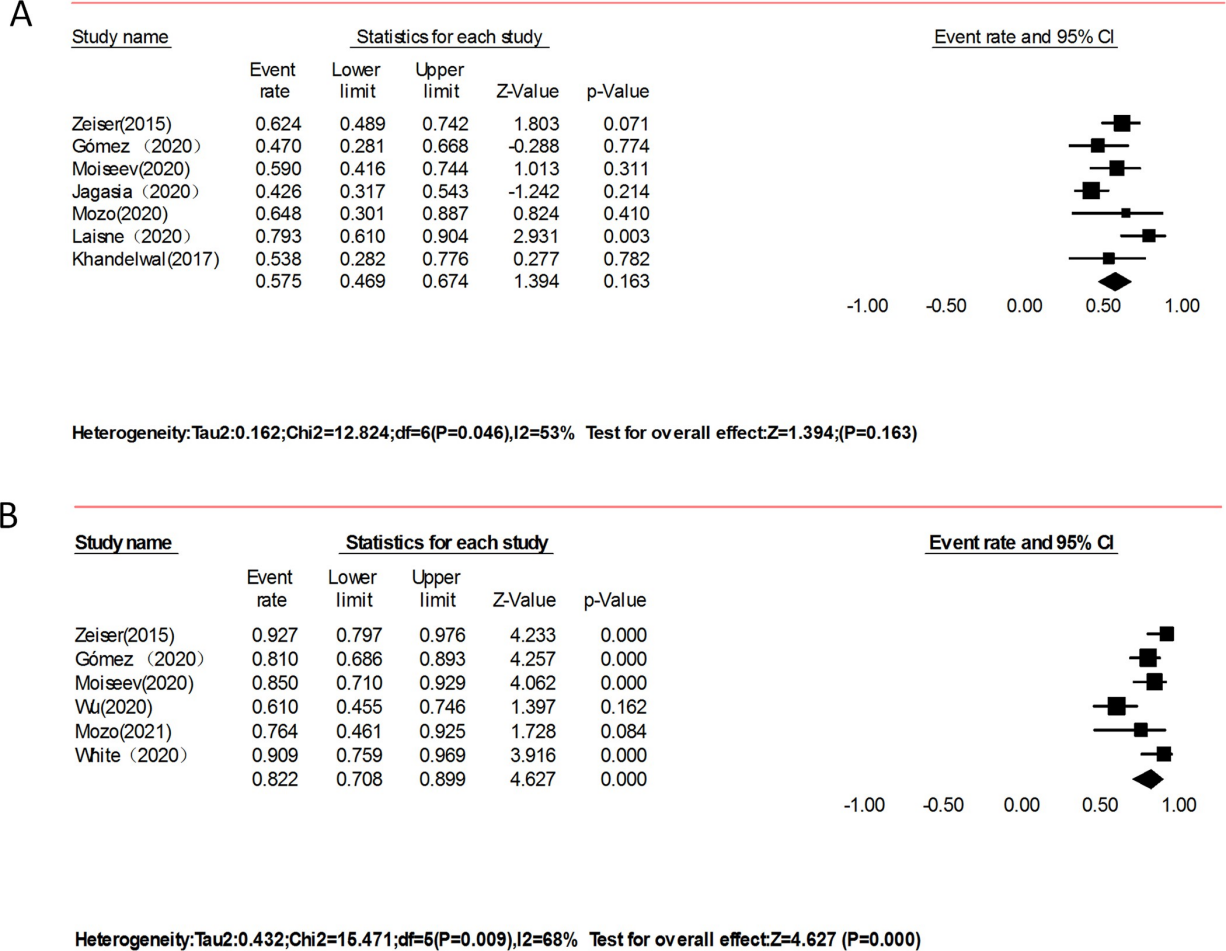
Forest plots of overall survival with 95% CI. Random effect models were used.

At the longest follow-up of the non-RCTs study, seven studies contained data for overall survival in 230 patients with aGVHD. Overall survival was 57.5% (95% CI 46.9–67.4, I^2^ = 53%; p = 0.046; Fig 3A). After removing two studies with apparent heterogeneity and sensitivity, the modified OS for aGVHD was 58% (95% CI 49.4–66.3, I^2^ = 0%). 82.2% (95% CI 70.8–89.9, I^2^ = 68%; p = 0.009; Fig 3B) in patients with cGVHD. The data were obtained from the other six studies, which included 228 patients. After removing one study with apparent heterogeneity and sensitivity, the modified OS for cGVHD was 85% (95% CI 79.0–89.8, I^2^ = 2.7%). In RCTs, the median overall survival for aGVHD was 11.1 months in the ruxolitinib group and 6.5 months in the control group (hazard ratio for death, 0.83; 95% CI, 0.60–1.15) [30]. The median overall survival in cGVHD was not reached in either group (hazard ratio, 1.09; 95% CI, 0.65 to 1.82) at the data cut-off [31].

#### Overall response (complete response or partial response)

**Fig 4:**
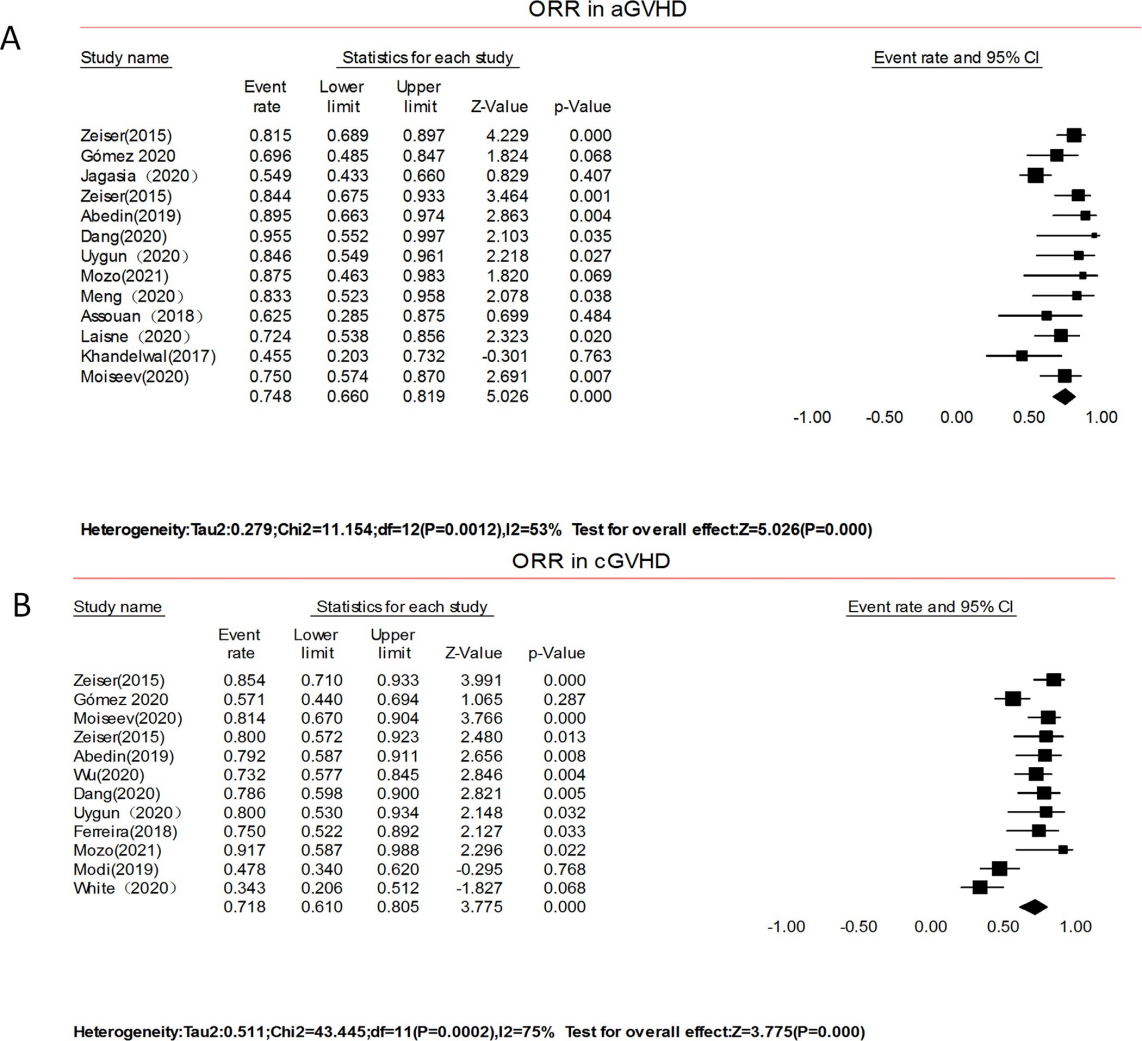
Forest plots of overall response.

The 13 Non-RCTs studies contained data for the overall response involving a total of 322 patients with aGVHD. The pooled proportion of ORR was 74.9% (95% CI 66.6–81.9, I^2^ = 53%, Fig 4A). After removing two studies with apparent heterogeneity and sensitivity, the modified ORR for aGVHD was 79% (95% CI 72.8–83.6, I^2^ = 0%). Four studies involving 61 children independently reported data on the response rate in aGVHD. The pooled ORR was 71.7% (51.9–85.6; I^2^ = 43%). The pooled proportion of ORR in adults with aGVHD was 73.1% (52.0–87.2; I^2^ = 77%), which was extracted from 4 studies (n = 152). A total of 381 patients with cGVHD were included in 12 non-RCT studies, which contained data on overall response. The pooled proportion of ORR was 73.1% (95% CI 62.5–81.6, I^2^ = 53%, Fig 4B). After removing three studies with apparent heterogeneity and sensitivity, the modified ORR for cGVHD was 79% (95% CI 73.8–84.1, I^2^ = 0%). Five studies involving 172 adults reported data on the response rate in cGVHD. The pooled ORR was 72.6% (56.7–84.3; I^2^ = 74%). In the RCTs studies, ruxolitinib significantly improved the overall response at 28 days of follow-up in aGVHD when compared with BAT (62% vs. 39%; odds ratio, 2.64; 95% CI 1.65–4.22; P<0.001) [30]. At week 24, patients with glucocorticoid-refractory cGVHD in the ruxolitinib arm had a better overall response than patients in the BAT arm (49.7% vs. 25.6%; odds ratio, 2.99; 95% CI, 1.86–4.80; risk ratio, 1.93; 95% CI, 1.44–2.60; P<0.001) [31].

#### Organ-specific response

**Fig 5:**
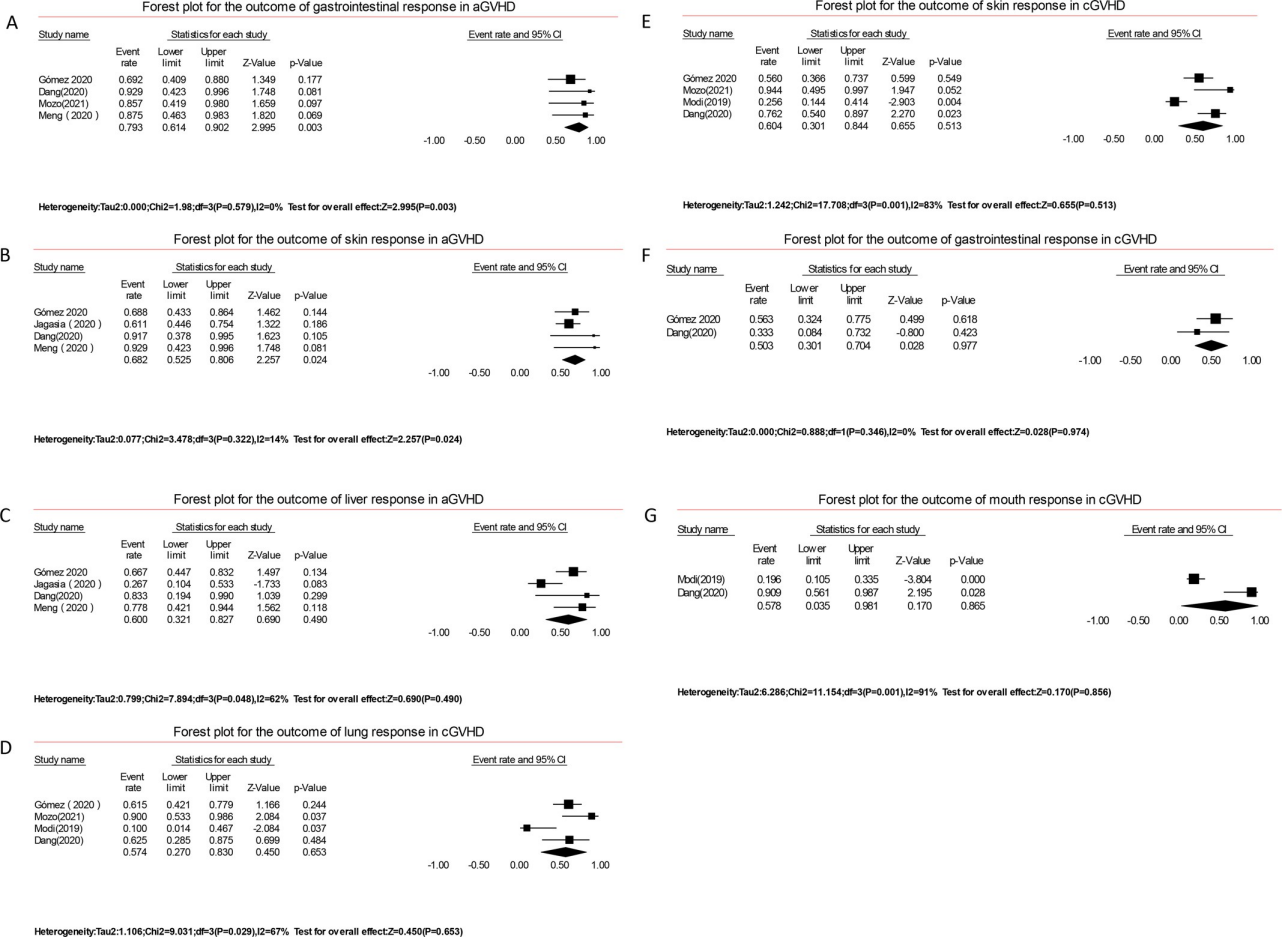
Forest plots of organ specific response (skin, liver, gastrointestinal, lung and mouth).

In terms of organ-specific response, 79.3% (61.4–90.2; I^2^ = 0%, Fig 5A) of patients had an ORR with gastrointestinal aGVHD in four studies involving 34 patients. Moreover, 68.2% (52.5–80.6; I^2^ = 14%, Fig 5B) of patients achieved an ORR with skin aGVHD in four studies involving 63 patients. 60% (32.1–82.7; I^2^ = 62%, Fig 5C) of patients had an ORR with acute liver GVHD in 4 studies involving 47 patients. For cGVHD, 60.4% (30.1–84.4; I^2^ = 83%, Fig 5E) and 57.4% (27.0–83.0; I^2^ = 67%, Fig 5D) ORR were achieved in the skin (n = 93) and lungs (n = 54) in four studies, respectively. In gastrointestinal GVHD 50.3%(30.1–70.4; I^2^ = 0%, Fig 5F) ORR was gained in 2 studies involving 22 patients. A total of 57.8% (3.5–98.1; I^2^ = 91%, Fig 5G) ORR was obtained in the mouth in 2 studies involving 57 patients. In all organ subgroups in REACH3, patients with glucocorticoid-refractory cGVHD in the ruxolitinib arm had a better overall response than those in the BAT arm (49.7% vs. 25.6%), particularly in the skin (52.9% vs. 25.7%; odds ratio, 3.24; 95% CI, 1.86–5.67) [31].

#### aGVHD vs. cGVHD

**Fig 6:**
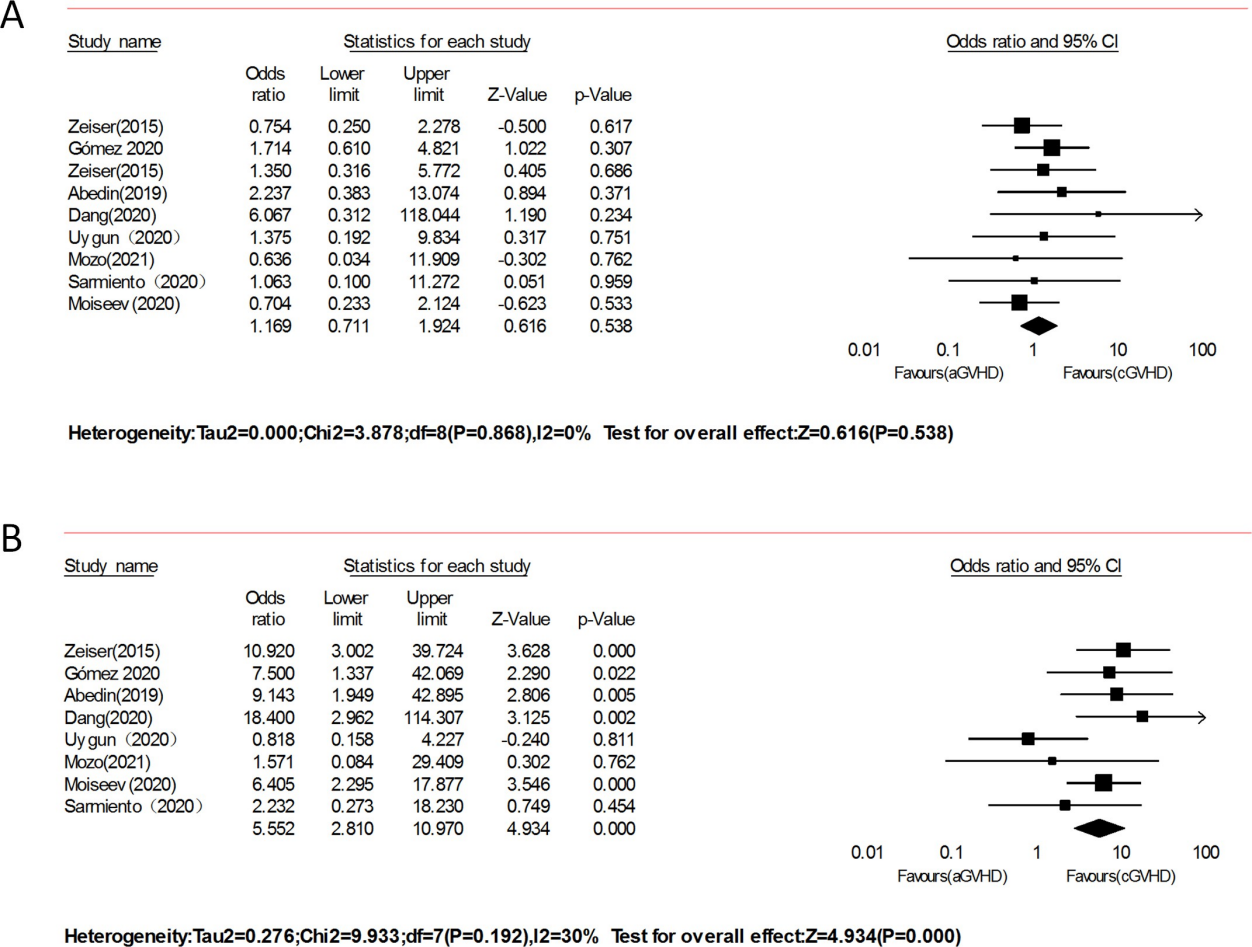
Forest plots of the overall response (ORR) of aGVHD vs. cGVHD after ruxolitinib therapy.

Under a random effects model, patients with SR-aGVHD showed a similar ORR (OR = 1.169, 95% CI: 0.711–1.924, p = 0.538, I^2^ = 0%, Fig 6A) in comparison with recipients with SR-cGVHD in 9 studies [13, 18, 21, 22, 24, 27–29, 32] involving 427 patients who were treated with RUX. In nine studies, only one study did not report the CR. Patients with aGVHD showed a better CR than patients with cGVHD (OR = 5.552, 95% CI: 2.81–10.97, p = 0.000, I^2^ = 30%, Fig 6B) in the remaining eight studies.

#### Grade II vs. grade III–IV

**Fig 7:**
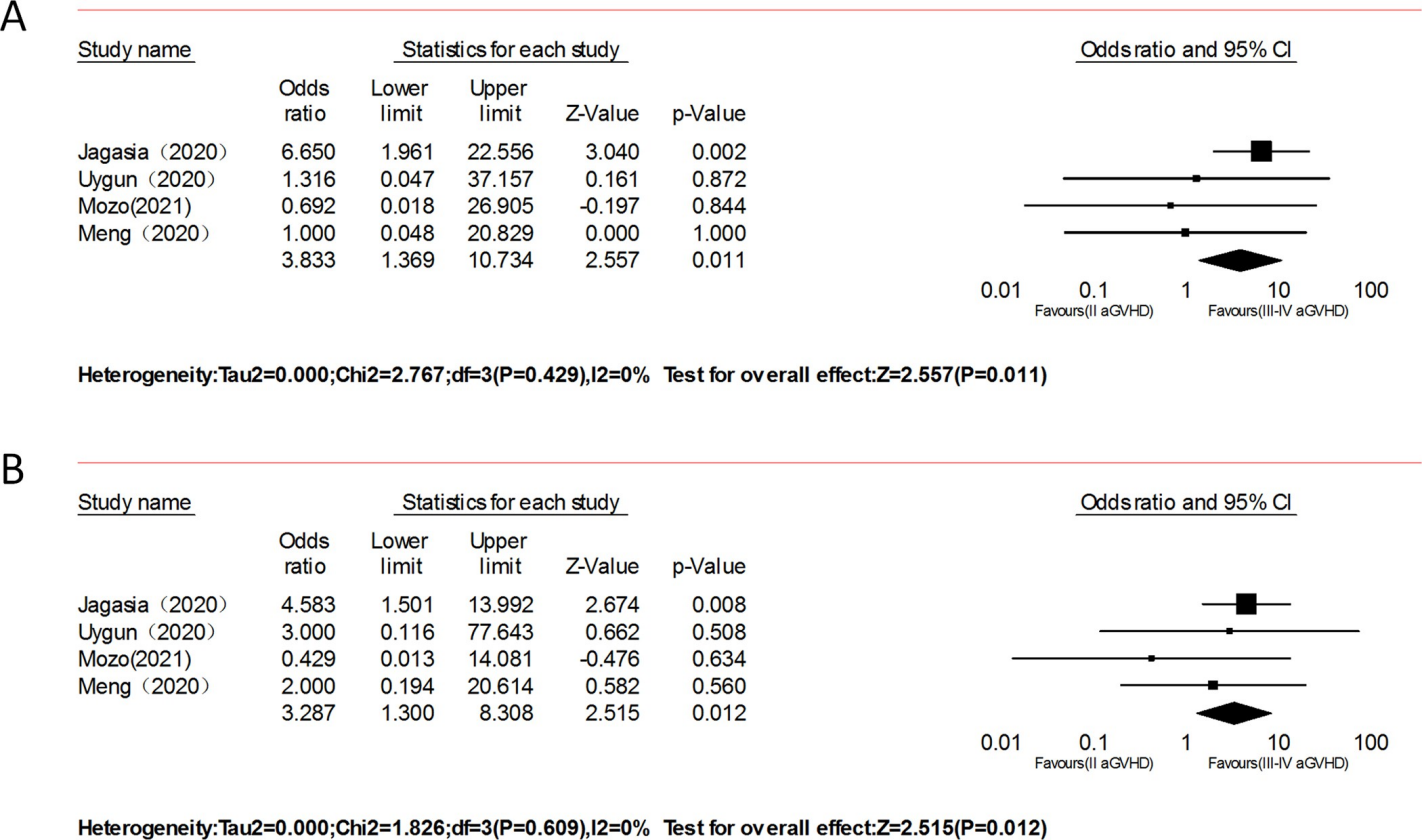
Forest plots of the overall response (ORR) of Grade II vs. Grade III–IV after ruxolitinib therapy.

Four studies involving 104 patients with aGVHD reported response rates [14, 16, 18, 21]. Patients with grade II SR-aGVHD showed a better clinical response than patients with grade III–IV SR-aGVHD (ORR: OR = 3.83, 95% CI: 1.37–10.73, p = 0.01, I^2^ = 0%, Fig 7A and CR: OR = 3.29, 95% CI: 1.30–8.31, p = 0.01, I^2^ = 0%; Fig 7B; respectively).

#### Moderate vs. severe

**Fig 8:**
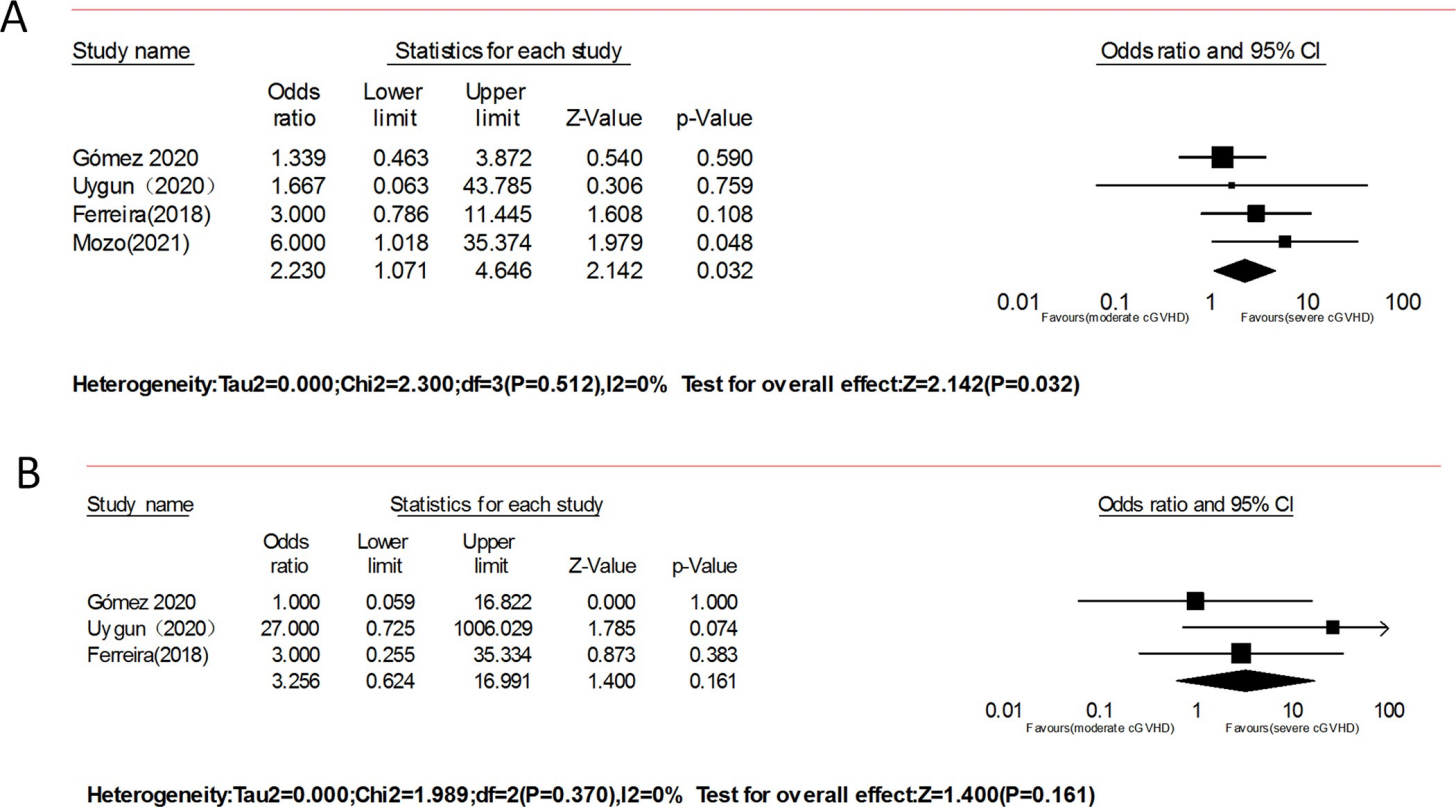
Forest plots of the overall response (ORR) of moderate vs. severe after ruxolitinib therapy.

Four studies [18, 19, 21, 29] involving 103 patients with moderate or severe SR- disease showed a better ORR (OR = 2.23, 95% CI: 1.07–4.65, p = 0.032, I^2^ = 0%, Fig 8A) after ruxolitinib therapy; after omitting one study without CR, the remaining studies had a similar CR (OR = 3.26, 95%CI: 0.62–17.00, p = 0.16, I^2^ = 0%, Fig 8B).

#### FFS and symptom response

Two randomised phase 3 trials (REACH2 and REACH3) evaluated failure-free survival [30, 31], and both demonstrated that patients receiving ruxolitinib had longer failure-free survival than patients receiving control therapy (median failure-free survival in aGVHD, >18.6 months vs. 5.7 months; HR, 0.37; 95% CI, 0.27–0.51; P<0.001); median failure-free survival in cGVHD, 5.0 months vs. 1.0 month; HR, 0.46; 95% CI, 0.35–0.60). In addition, the response to the modified Lee Symptom Scale was only described in patients with glucocorticoid-refractory cGVHD [31]; the ruxolitinib arm at 24 weeks had a higher response than the control group (24.2% vs. 11.0%; OR, 2.62 [95% CI, 1.42 to 4.82]; RR, 2.19 [95% CI, 1.31–3.65]; P = 0.001).

#### Adverse events

Adverse events developed in most patients in all studies. The most common haematological adverse event was cytopenia, including thrombocytopenia, anaemia, and leukopenia. The most common non-haematological adverse event was infectious complications (>60%). Regarding the types of infections, cytomegalovirus (CMV) reactivation is a remarkable risk, and the incidence of reactivation varies from 20% to 50% among patients. Bacterial and fungal infections are also common. It would be inappropriate to conduct a meta-analysis of adverse events because of the variation in different studies (such as study types and drug dose).

#### Sensitivity analyses

We performed a sensitivity analysis with OS and ORR after removing studies with apparent heterogeneity and sensitivity. The modified OS and ORR were similar to those obtained in the initial assessment.

#### Publication bias

**Fig 9:**
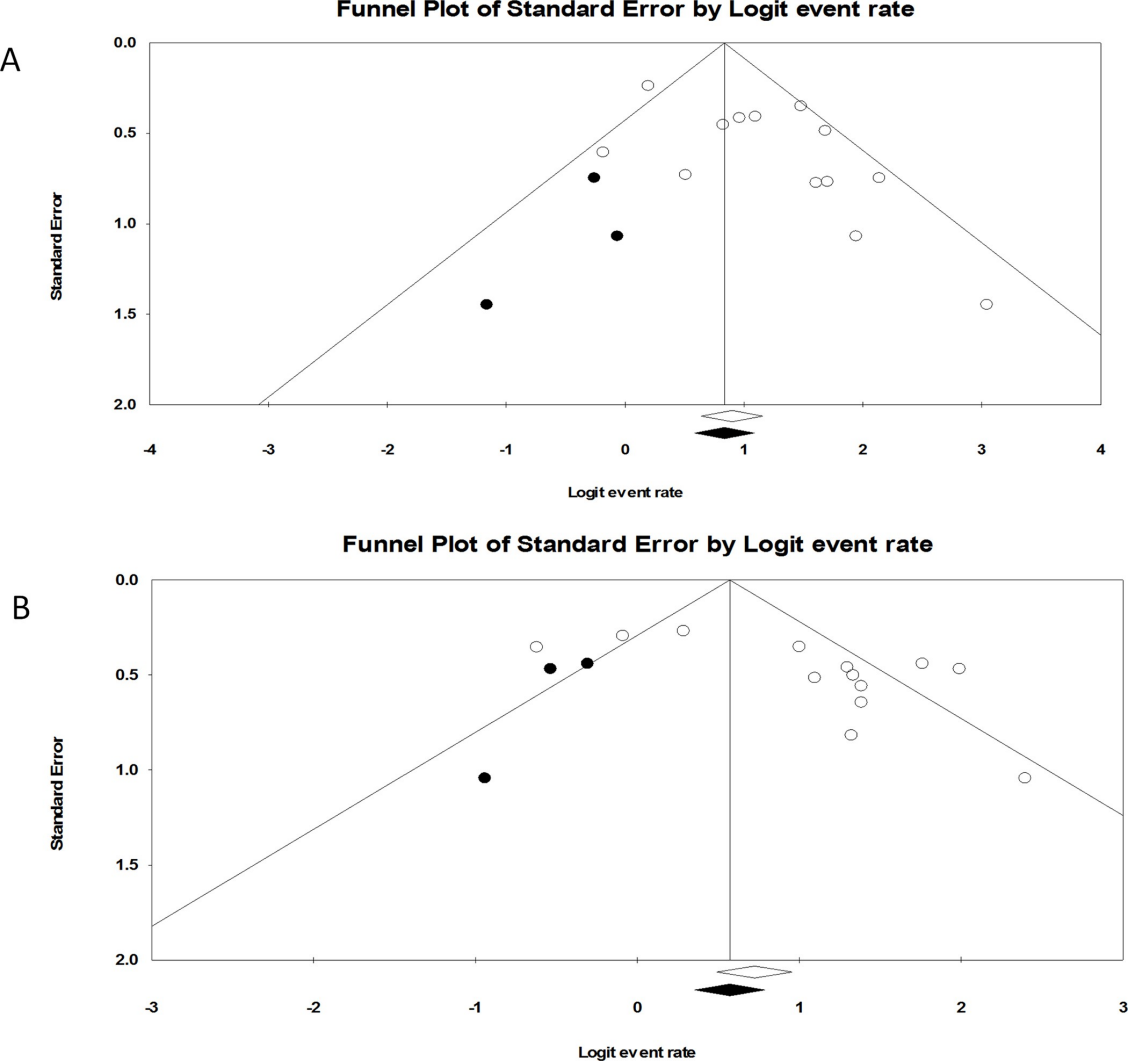
Adjusted funnel plot after trim and fill indicated the exit of publication bias.

The potential publication bias of ORR in non-RCT studies was assessed in this meta-analysis. By comparing the funnel plots, we found a publication bias. Additionally, the Egger test showed an asymmetric distribution (P = 0.03 aGVHD; P = 0.02 cGVHD), indicating the existence of publication bias. The adjusted funnel plot after trim-and-fill indicated that the estimated effect with the addition of three studies was 0.721 (95% CI: 0.631–0.795, Fig 9A) in aGVHD and 0.669 (95% CI: 0.564–0.759, Fig 9B) in cGVHD. The unadjusted estimated effects were 0.748 (95% CI: 0.660–0.819) and 0.718 (95% CI: 0.610–0.805), which also demonstrates the existence of publication bias. Therefore, the results of this meta-analysis may have overestimated the efficacy of ruxolitinib. However, the results should be explained carefully because of differences in methodological quality between studies, unpublished negative results, and sample sizes.

## Discussion

SR-GVHD remains a serious clinical problem. The results of our meta-analysis show that ruxolitinib therapy affects overall survival in patients with SR-GVHD, especially SR-cGVHD. The ORR was similar and was more than 70% in both acute and chronic GVHD. Gastrointestinal aGVHD, lower aGVHD grade, and moderate cGVHD were associated with better clinical response. Moreover, the studies included in the meta-analysis reported a higher risk of infection and cytopenia, which seemed manageable. Additionally, the relatively manageable toxicities indicated that ruxolitinib was safe for the treatment of patients with SR-GVHD.

Studies in mice and humans have indicated that tissue damage releases interleukin-1β and interleukin-6, which induces Th17-cell differentiation, promote the development of chronic GVHD. Moreover, dysregulated B-cell and T-cell, including regulatory B, regulatory T, follicular regulatory T, type 1 regulatory T, type 2 helper T, interferon-γ-producing type 1 helper T, and invariant natural killer T cells, are associated with the development of cGVHD. Last, tissue fibrosis and macrophage activation may contribute to the development of cGVHD [6, 33, 34]. Interactions between T cells and antigen presenting cells expressing host major or minor histocompatibility antigen peptides and co-stimulation can cause GVHD. Inflammatory mediators, such as interleukin-33, interleukin-1β, type 1 helper T cell cytokines (interferon-γ, interleukin-2, TNF), and reactive oxygen species, were also correlated with acute GVHD [35, 36].

The standard first-line treatment for GVHD is corticosteroids. However, there is no consensus regarding the treatment of SR-GVHD. In one meta-analysis involving MSCs [37], 50% of MSC-treated patients were alive at the longest follow-up (63% of patients were alive at 6 months), 72% of patients had an overall response. In another meta-analysis [38], the anti-CD20 monoclonal antibody rituximab showed a 66% ORR, and skin GVHD had a better clinical response. One systematic review [39] evaluated the efficacy of ECP for the treatment of steroid-refractory or steroid-dependent GVHD; the ORRs were 69% and 64% for acute and chronic GVHD, respectively. Our findings are similar to those of a previously published systematic review and meta-analysis [40] that included 16 studies involving 414 patients, which had an ORR of 77% and 81% in acute and chronic SR-GVHD, respectively. The 1-year OS rates were 60% and 89% for aGVHD and cGVHD, respectively, in adults. Moreover, compared to that meta-analysis, we included new studies and excluded those with fewer than 10 patients. Two important large RCT trials were published subsequent to the previous reviews, which allowed us to reanalyse the effect of ruxolitinib on ORR and OS. In non-RCTs, 57.5% and 82.2% of the patients were alive at the longest follow-up. The ORRs were 74.9% and 73.1% in acute and chronic GVHD, respectively. Two large randomised phase 3 trials (REACH2 and REACH3) [30, 31] showed that patients with glucocorticoid-refractory GVHD in the ruxolitinib arm had a better overall response compared with patients in the BAT arm (OR in aGVHD at 28 days: 62% vs. 39%; odds ratio, 2.64; 95% CI 1.65–4.22; P<0.001 and OR in cGVHD at 24 weeks: 49.7% vs. 25.6%; odds ratio, 2.99; 95% CI, 1.86–4.80; risk ratio, 1.93; 95% CI, 1.44 to 2.60; P<0.001).

Ruxolitinib impairs dendritic cell and T-cell functions, leading to imbalances in cytokine production. This phenomenon increases the risk of opportunistic infections [41]. JAK2 plays an important role in erythropoietin and thrombopoietin signalling [42]. JAK-signal transducers and activators of transcription pathway cytokine-mediated haematopoiesis. Cytopenia was the main adverse event associated with ruxolitinib therapy. Moreover, close monitoring for CMV reactivation and appropriate prophylaxsis is required on treatment with ruxolitinib.

This meta-analysis had several limitations. First, some studies had small sample sizes. Second, the treatment for SR-GVHD is similar, but the clinical and pathophysiological features between aGVHD and cGVHD are different essentially. Thus, it is not certain whether the comparison between aGVHD and cGVHD in ORR or CR is meaningful. In addition, the diagnostic criteria and definitions for SR-GVHD varied across studies, which may make the comparison of results less compelling. Third, there were some differences between the assessed studies, including drug dose, initial diagnosis, donor and recipient HLA matching degree, conditioning regimen, immunosuppressive drugs, sample size, age, and gender. All these factors may have contributed to the heterogeneity observed between the studies. Last but not the least, we could not evaluate dose effects, which were not reported in all studies. In addition, adverse events could not be evaluated individually due to differences in reporting in the original studies (e.g., “infection” “Cytopenia”).

Our systematic review and meta-analysis indicated that ruxolitinib therapy may be more effective in patients with low-grade aGVHD and gastrointestinal involvement. In addition, two high-quality, large-scale RCTs with 638 patients demonstrated the efficacy and safety of ruxolitinib in treating SR-GVHD, which is an advantage for second-line therapy in these cases.
